# Automated analysis of digital fundus autofluorescence images of geographic atrophy in advanced age-related macular degeneration using confocal scanning laser ophthalmoscopy (cSLO)

**DOI:** 10.1186/1471-2415-5-8

**Published:** 2005-04-06

**Authors:** A Deckert, S Schmitz-Valckenberg, J Jorzik, A Bindewald, FG Holz, U Mansmann

**Affiliations:** 1 Institute of Medical Biometry and Informatics (IMBI), University of Heidelberg, Im Neuenheimer Feld 305, 69120 Heidelberg, Germany; 2 Department of Ophthalmology, University Eye Hospital, Abbe-Straß2, 53127 Bonn, Germany; 3 Department of Ophthalmology, University Eye Hospital, Im Neuenheimer Feld 400, 69120 Heidelberg, Germany; 4 Institute of Medical Information, Biometry, and Epidemiology (IBE), University of Munich, Marchioninistrasse 15, 81377 Munich, Germany

## Abstract

**Background:**

Fundus autofluorescence (AF) imaging using confocal scanning laser ophthalmoscopy (cSLO) provides an accurate delineation of areas of geographic atrophy (GA).

Automated computer-assisted methods for detecting and removing interfering vessels are needed to support the GA quantification process in longitudinal studies and in reading centres.

**Methods:**

A test tool was implemented that uses region-growing techniques to segment GA areas. An algorithm for illuminating shadows can be used to process low-quality images. Agreement between observers and between three different methods was evaluated by two independent readers in a pilot study. Agreement and objectivity were assessed using the Bland-Altman approach.

**Results:**

The new method (C) identifies vascular structures that interfere with the delineation of GA. Results are comparable to those of two commonly used procedures (A, B), with a mean difference between C and A of -0.67 mm^2 ^(95% CI [-0.99, -0.36]), between B and A of -0.81 mm^2^, (95% CI [-1.08, -0.53]), and between C and B of 0.15 mm^2 ^(95% CI [-0.12, 0.41]). Objectivity of a method is quantified by the mean difference between observers: A 0.30 mm^2 ^(95% CI [0.02, 0.57]), B -0.11 mm^2 ^(95% CI [-0.28, 0.10]), and C 0.12 mm^2 ^(95% CI [0.02, 0.22]).

**Conclusion:**

The novel procedure is comparable with regard to objectivity and inter-reader agreement to established methods of quantifying GA. It considerably speeds up the lengthy measurement process in AF with well defined GA zones.

## Background

Age-related macular degeneration (AMD) is the most common cause of legal blindness among industrialized nations in the population aged 50 years and above [1-4]. Besides choroidal neovascularization and detachments of the retinal pigment epithelium, geographic atrophy (GA) of retinal pigment epithelium (RPE) is a common cause of severe visual loss in patients with AMD [5-7]. Changes in time can be documented by fundus autofluorescence images (AF) mediated by RPE lipofuscin accumulations and its spatial distribution over retinal areas, obtained in vivo using a confocal scanning laser ophthalmosocope (Heidelberg Retina Angiograph (HRA), Dossenheim, Germany) [8-10]. Areas of GA are usually associated with a well-defined zone of decreased autofluorescence due to the absence of fluorophores residing in RPE lipofuscin granules [11,12]. The deduction of clinically relevant information from these pictures is a complex process which should be optimised and automated, especially in the context of multicenter studies.

Generally, AF are recorded using a confocal scanning laser ophthalmoscope (Heidelberg Retina Angiograph, HRA, Heidelberg Engineering, Germany; which includes the Heidelberg Eye Explorer (HEE) software package). The images are immediately digitised and processed using a flexible frame processor and subsequently displayed on a computer screen. Corresponding with funduscopically visible atrophic areas, fundus intensity of AF is markedly decreased [13].

With method A, atrophic areas are outlined on the screen using the mouse-driven cursor of the HEE software program. The areas are then measured and the data exported manually to an Excel spreadsheet by cut and paste. This completely manual, mouse-driven method A is time-consuming and can exaggerate subjective impressions. Mistakes can occur as a result of the error-prone interface between the user's hand and the computer mouse [13]. This implies that the accuracy of mouse-driven contour painting depends not only on subjective impressions, but also on the user's dexterity.

With method B, the images are exported as bitmap files from the HEE program. Interfering vascular structures, which appear as dark atrophic areas, are manually repainted white using the mouse-driven paintbrush of Microsoft Paint. The modified images are then transferred to Global Lab Image 2 and the remaining dark areas are measured using a threshold procedure tool. The resulting data are exported to Microsoft Excel manually by cut and paste [13]. This semi-automated method B only allows an interpretation using high-quality AF images. It requires the manual, mouse-driven whitewashing of interfering vessels that touch upon, or extend into, the GA area. However, as the vessels exhibit grey levels that are similar to atrophic patches, automated segmentation is not possible [13]. Both methods rely on the circumstantial handling of different software tools that have not been specifically adapted to the problems of GA measurements.

We have developed a novel customized image analysis test tool that includes an adapted algorithm for automated identification of interfering vascular structures, and compared this new method with the previous ones.

## Methods

The test tool combines all steps of GA area measurement, including the automatic export of data into an Excel spreadsheet for further analysis. The method is based on region-growing, instead of segmentation with a threshold value similar to that of method B, allowing the reader to sort out non-atrophic areas with similarly diminished grey values as in actual GA areas.

After selecting the GA area by moving the mouse cursor and clicking on the region of interest, a first segmentation is started using a default parameter value calculated by the image's mean grey value. Automatic correction of the generated contour is possible by adjusting the associated parameter value with a relocatable graphical element. Holes within the detected GA area can be identified and further GA areas in the same image can be integrated.

For segmentation of GA areas with interfering vascular structures, the tool includes an optional algorithm for detecting and whitewashing such structures [14]. The number of detected vessels depends on the individual settings of the parameters for vessels diameter, length and cross-linking. Incorrect contour segments of the GA caused by whitewashed vessel stubs can reach into the GA area and are corrected, either automatically with a default parameter or by user interaction.

The algorithm eliminates vessel stubs within the GA area and corrects the contour segments in these regions. One disadvantage of this process is that small contour jags which were correctly detected a step before now disappear, and instead the contour is minimally smoothed and widened. As a result the process produces correct contour segments in passages from GA area to vessels and incorrectly widened contour segments otherwise. A new segmentation step produces the correct contour without interfering vessels.

Using well-aligned relocatable convex and radial hulls facilitates fine-tuning of the actual delineation and measurement of GA areas. These tools enable alternative contour finding in difficult contour segments with tangentially interfering vessels. Poor quality images often contain large shady outer areas causing erupting segmentation of GA areas before identifying the true borders of the GA. This can be countered by the method's option for illuminating such areas of shade. The original image is shown in a separate window. An electronic magnifier is integrated to support contour finding in difficult sections as well as for small GA areas.

### Validation

Validation is based on the material collected in a previous study [13], with published data from the right eye only. Data for both eyes are available [13] and are used for validation. The same readers evaluated the same material, but using the new method. The images were evaluated in random order and the readers had no access to the previous results based on methods A and B. With the unpublished material from 2001, our validation sample consists of 10 left and 24 right eyes. The Bland-Altman Design for method comparison studies [15] was applied. The limits of agreement (REF) were calculated for each comparison. Nonparametric tests for paired and unpaired observations as well as ANOVA (Analysis of Variance) were used for statistical assessment. The significance level was set to α = 0.05.

The study was a sub-study of the FAM study which followed the tenets of the Declaration of Helsinki and was approved by the Ethics Committee of the University of Heidelberg. Informed consent was obtained from the patients prior to recruitment into the study.

## Results

**Figure 1:**
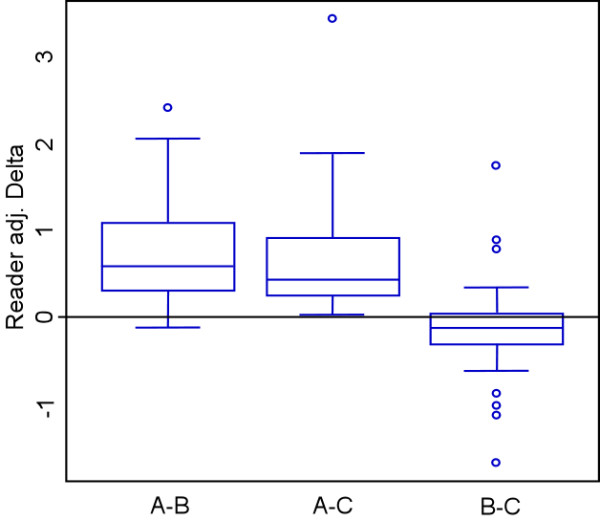
**Box-plot method comparison. **Reader adjusted mean difference in the quantified GA area [mm^2^] between methods: A-B, A-C, B-C.

**Figure 2:**
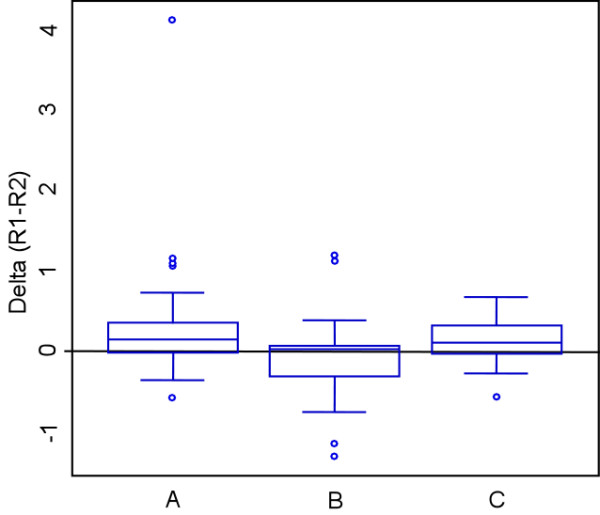
**Box-plot reader comparison. **Method induced mean difference [mm^2^] between two readers.

Table 1 shows the results for the 40 eyes included in the study. Method C produced values which lay between those of methods A and B. The quantification of the agreement following Bland and Altman [15] between methods B and A, C and A, and B and C is given in the upper half of table 2 (mean difference, 95% confidence interval, REF). Differences in the quantified area are presented as box-plots in figure 1 and figure 2. Using the Friedman test [16], a global distinction between methods could be shown for each of the readers (reader 1 p < 0.001, reader 2 p < 0.001). The Wilcoxon test gives significant distinctions for reader 2 between method A and B (p < 0.001) and between A and C (p < 0.001). No significant difference could be found between methods B and C (p = 0.467). For reader 1, the results were similar, except that methods B and C also do not differ significantly (A/B p < 0.001; A/C p < 0.001; B/C p = 0.027). The investigation of objectivity by the Bland-Altman design is given in the lower half of table 2 (mean difference, 95% confidence interval, REF). The box plots presented in figure 2 show a slightly larger bias between the readers for method C in comparison with method B and less bias in comparison with method A. Differences between the readers is established for all three methods by the Wilcoxon test (A: p = 0.007; B: p = 0.012; C: p = 0.018). Combining agreement between readers, the Friedman test shows global significant distinctions between all methods (p < 0.001). An analysis of variance provided a deeper insight into the cause of the observed reader effect, which is not homogeneous for all methods. The ANOVA approach establishes an interaction between both effects. The significant interaction term (p < 0.01) demonstrates the clear dependence of a method's result on the reader using it.

## Discussion

Comparability, repeatability, and objectivity are crucial factors to consider when developing a new method. Comparability with at least two methods has to be determined; objectivity calls for a multitude of readers; and repeatability requires multiple measurements by the same reader using each method. An appropriate study would randomly allocate image and reader in a cross-over design.

Bland-Altman plots visualize comparability, repeatability, and objectivity in terms of the limits of agreement in which 95% of data should appear. Furthermore, the data within these borders must lie within the region of clinically irrelevant differences. Therewith, verification of a clinically comparable quality can be obtained. The measurements for method comparison have to be performed at the same time to exclude subjective effects caused by changing user criteria rather than by the methods themselves.

Additionally, improved effectiveness must be demonstrated to justify replacement of methods [15]. Effectiveness is defined here as the number of successfully assessed AF images and the time required to complete the assessment procedure. In 2001 [13] no exact measurements of reading duration were taken. However, both readers had the impression that method C speeds up the reading process. Furthermore, low-quality images were excluded from the readings in 2001 [13]. Some of these pictures could be handled with method C. Again both readers agreed that method C was better suited to evaluate low-quality images. A further study has to put these subjective impressions into an appropriate objective framework to demonstrate effectiveness.

We conclude that method C is not inferior to the two commonly methods used in measuring GA areas. This has been shown by the comparability of the measured values in the Bland-Altman design. Thereby, the inter-methods comparisons align with the degree of decision freedom for each method. Method A [13] enables the setting of each contour pixel individually with no relation between contour pixels. Method B [13] has only one degree of decision freedom using one threshold value for the whole contour, and method C has fewer degrees of decision freedom than A, but more than B: it allows for the individual exclusion of non-atrophic holes or a fine-tuning of critical contour-sections. In accordance with the degrees of decision freedom, the mean values of the new method C lie between those of method A and B (data not shown). Altogether, method C is similar more to method B than to method A (see table 2, A-C, B-C). Increasing the number of degrees of decision freedom increases the influence of subjectivity. It also gives a larger impact of user's competence.

**Figure 3:**
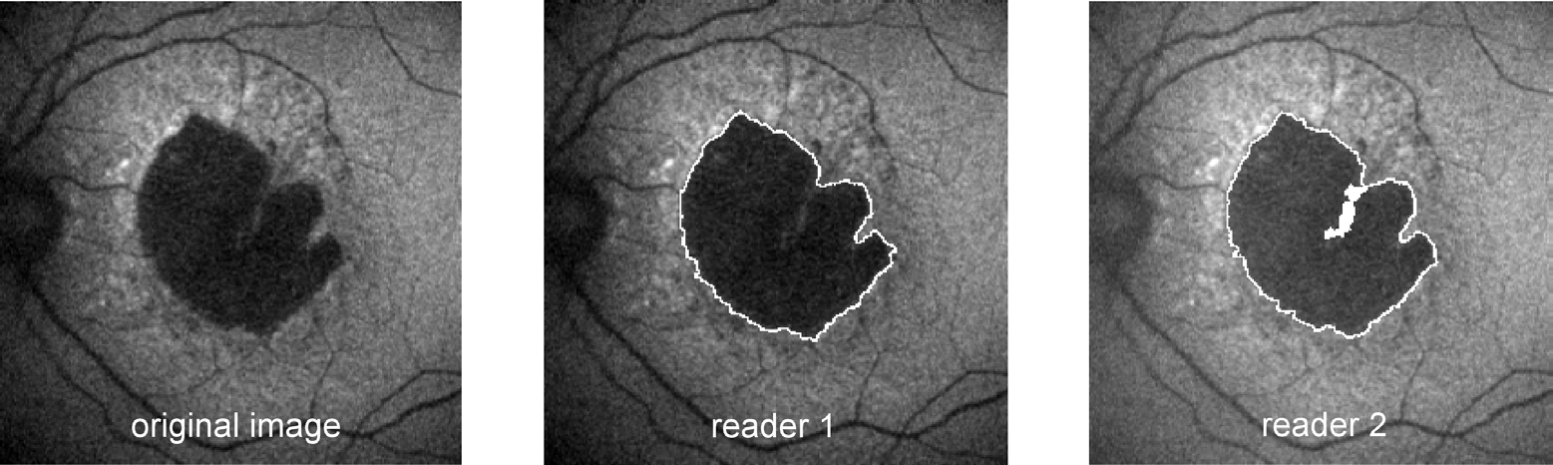
**Example for different interpretation with method C. **Reader 1 defines a non-atrophic area within the GA in a second step (filled white area) which will not be considered for the calculation of the area size.

**Figure 4:**
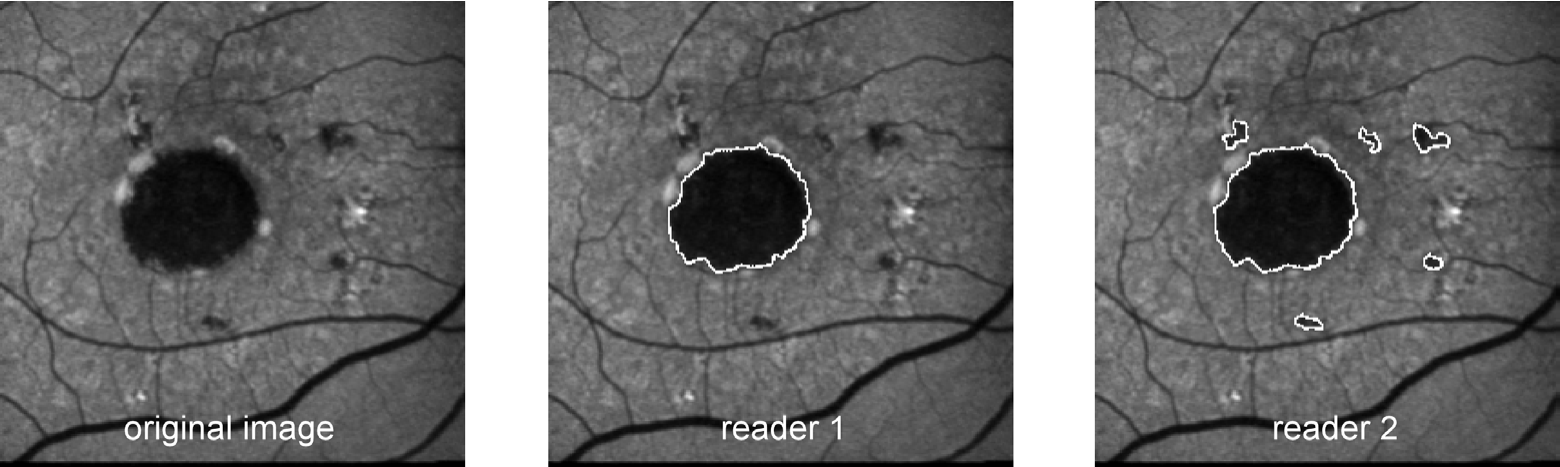
**Example for different interpretation with method C. **This example shows differences in the perception of what is a GA. Reader 2 declares additionally small GA spots.

Method B has only one degree of decision freedom and should theoretically be the most objective. But the bias in objectivity of method C is, first of all, a result of more than one degree of decision freedom (in comparison with method B). Therefore, the readers were trained in using method C. This made the objectivity of method C not significantly inferior to method B. The remaining bias of method C is redeemed by fewer outliers and less dispersion. Even within method C, there was a clear difference in how the readers interpreted the same AF images (see figure 3 and figure 4).

There are subjective inter-reader effects and effects caused by changing assessment criteria within the same reader over a period of years due to increasing experience or changing criteria, but not due to the method used. This influences the bias in method comparison and therewith the statements about measurement error. Considering this confounder, method comparison can only show that method C is no worse than methods A and B. The effect of the influence by the subjectivity of readers is most distinct in method A (see table 2).

Heterogeneity in agreement between the readers over the methods was demonstrated by a significant interaction term (reader by method) in an ANOVA model. There is a loss of histological information due to the process of producing AF images from the retina by confocal laser scanning ophthalmoscopy. Several underlying factors for alterations in grey values in the AF images, mainly in the border region of GA areas, force the reader to interpret by his subjective impressions, more or less supported by the interaction between him and the method.

## Conclusion

The study is a pilot study, yet it has provided important information for the further design and testing of method C. Method C has been developed and launched in consideration of several viewpoints. One aim has been to reduce the influence of manual skills and the number of procedural steps, while another important goal was not to restrict the competence of the medical user.

Method C can be implemented to quickly assess both unproblematic AF images and, with the additional accessory tools, difficult AF images. For example, the presence of shadows within the marginal areas of the images appears to be the main reason for poor quality. Presenting a processed image with illuminated shadows together with the original image facilitates segmentation by different algorithms. Furthermore, robust algorithms have been integrated to rectify segmentation and to eliminate interfering vessels in an effective way. Hence, a large proportion of GAs can be measured in a short time using vessel detection and default parameters.

The individual nature of each AF image and the wide variation of possible combinations of features suggest that it would be impossible to develop a clearly defined method that could measure GA areas with only a few procedural steps. Thus, it seems to be recommendable to represent in the future a combination of several methods, with adaptations depending on the quality of AF images, in one tool. However, in attempting to decide upon a particular combination of method parts, some basic questions arose during the process of developing and testing method C, which have still to be clarified.

The selection of further algorithms and methods will depend on the definition of the clinical relevance of differences between GA areas. Consequently, care should be taken that no clinical relevance is attached to artificial differences due solely to repeated use of a method or to different users. Furthermore, a method comparison using the Bland-Altman design will only be meaningful if there are well-defined limits of agreement and clinically relevant bounds [15]. For a method to be useful, the probability of differences between repeated measures and objectivity must not transgress this bound significantly. If two methods produce absolute differences below this limit with non-significant probabilities, then the method with low dispersion but nearer the bound should be preferred. It should be favoured over a method with less bias but larger dispersion. The reason is that a uniform bias could be eliminated by training or considered in the statistical evaluation. With regard to objectivity, it is also important to know whether high objectivity by one threshold value like in method B limits precise contours and facilitates medically correct detection of GA borders. If the precision in imaging the GA borders is important for quantification of disease progression, a segmentation algorithm like region-growing with more degrees of decision freedom should be used. For the decision, clinical relevance in method comparison should be the main criteria, too.

**Figure 5:**
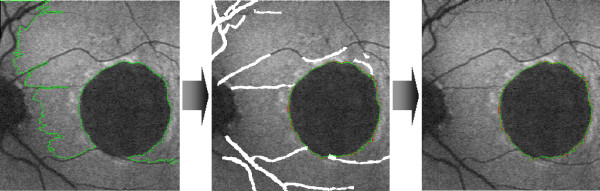
**Example for image processing. **Interfering vessels prevent correct segmentation as shown in the first image. Whitewashing the interfering vessels allows a correct segmentation of the GA.

We conclude with an example of successful segmentation after whitewashing the vessels, presented in figure 5. The first segmentation produced erupted contours (first image, green line) caused in each case by interfering vessels. After automated whitewashing with corrected GA entries of white vessel stubs the accurate GA contour is found (second image, green line). Fine tuning of this contour within the wider red one is possible.

## Competing interests

The author(s) declare that they have no competing interests.

## Authors' contributions

AD has developed the new method, performed the study and composed the manuscript. SS and JJ performed the evaluation of the pictures in the validation study. AB and FH have given support in ophthalmologic issues and have provided patients data and ophthalmologic devices. UM initialised and supervised the project, he also contributed to the manuscript.

## Pre-publication history

The pre-publication history for this paper can be accessed here:



## Figures and Tables

**Table 1:** The complete GA measurement data set

**method**	**reader 1 **(mm^2^)			**reader 2 **(mm^2^)		
	**A**	**B**	**C**	**A**	**B**	**C**
**ID right eyes**						
7	8,86	8,335	8,737	8,94	8,286	8,711
8	8,08	5,209	6,913	7,01	5,759	6,34
12	10,17	9,42	9,372	9,73	9,368	9,965
13	10,1	9,163	9,462	10,48	9,228	9,174
16	21,58	19,821	21,093	21,44	20,407	20,908
28	2,65	2,352	2,15	2,49	2,29	2,44
29	9,47	8,861	9,163	9,5	9,07	8,672
53	7,17	6,606	6,864	7,04	6,595	6,688
65	11	9,868	10,226	10,82	9,488	10,316
67	2,22	2,029	2,193	2,19	2,029	2,143
68	12,07	10,916	11,218	11,54	10,785	10,874
89	12,23	11,3	10,466	11,1	10,117	10,259
90	4,36	4,095	4,174	4,63	4,339	4,114
96	10,82	8,832	10,312	10,1	10,18	9,947
97	27,1	23,976	23,533	22,95	22,868	23,769
102	23,69	20,433	21,489	22,6	21,023	21,05
109	10,7	9,032	10,153	10,39	9,112	10,04
113	12,01	11,115	11,283	11,67	10,911	11,384
144	3,86	3,555	3,576	3,84	3,653	3,649
146	2,95	2,695	2,718	2,97	2,677	2,764
147	1,8	1,504	1,478	1,92	1,504	1,449
167	2,82	2,396	2,438	3,01	2,396	2,62
176	4,03	3,671	3,936	3,98	3,677	3,869
182	1,68	1,328	1,433	1,69	1,418	1,484

**ID left eyes**						

7	23,54	22,78	22,186	23,38	22,993	22,026
8	15,23	12,86	15,228	14,8	13,644	14,661
12	3,2	2,795	2,968	2,94	2,793	2,662
13	12,78	12,682	12,588	13,4	13,009	12,501
16	24,34	22,687	21,023	24,43	22,677	20,855
29	10,3	9,139	8,65	10,35	9,477	8,201
67	0,66	0,536	0,512	0,6	0,965	0,528
89	11,62	11,156	11,459	11,53	10,954	10,798
99	11,62	10,887	11,259	11,46	10,826	11,001
120	6,23	4,594	4,856	6	5,781	5,15

**ID in 2001 excluded eyes**						

77			6,843			6,783
86			7,665			6,977
92			21,719			21,223
123			7,038			7,005
161			4,67			4,475
186			5,474			5,254

**Table 2:** Quality measures to assess method comparison

		Mean difference (mm^2^)	95% confidence interval	Limits of agreement
Agreement	A-B	0.81	[-0.53; 1.08]	[-0.76; 2.37]
	A-C	0.67	[0.36, 0.99]	[-1.13; 2.47]
	B-C	-0.15	[-0.41; 0.12]	[-1.68; 1.38]

Objectivity	A	0.30	[0.02; 0.57]	[-1.27; 1.86]
	B	-0.11	[-0.28; 0.07]	[-1.10; 0.88]
	C	0.12	[0.02; 0.22]	[-0.44; 0.68]
